# Galbase: a comprehensive repository for integrating chicken multi-omics data

**DOI:** 10.1186/s12864-022-08598-2

**Published:** 2022-05-12

**Authors:** Weiwei Fu, Rui Wang, Naiyi Xu, Jinxin Wang, Ran Li, Hojjat Asadollahpour Nanaei, Qinghua Nie, Xin Zhao, Jianlin Han, Ning Yang, Yu Jiang

**Affiliations:** 1 Key Laboratory of Animal Genetics, Breeding and Reproduction of Shaanxi Province, College of Animal Science and Technology, Northwest A&F University, Yangling, 712100 China; 2 Department of Animal Genetics, Breeding and Reproduction, College of Animal Science, South China Agricultural University, Guangzhou, 510642 Guangdong China; 3 Department of Animal Science, McGill University, Montreal, Québec Canada; 4 CAAS-ILRI Joint Laboratory On Livestock and Forage Genetic Resources, Institute of Animal Science, Chinese Academy of Agricultural Sciences (CAAS), Beijing, China; 5 Livestock Genetics Program, International Livestock Research Institute (ILRI), Nairobi, Kenya; 6 National Engineering Laboratory for Animal Breeding and Key Laboratory of Animal Genetics, Breeding and Reproduction, Ministry of Agriculture and Rural Affairs, China Agricultural University, Beijing, 100193 China; 7 Center for Functional Genomics, Institute of Future Agriculture, Northwest A&F University, Yangling, China

**Keywords:** Chicken, Omics data, Complex traits, Database

## Abstract

**Background:**

Multi-omics data can provide a stereoscopic view to explore potential causal variations and genes, as well as underlying genetic mechanisms of complex traits. However, for many non-mammalian species, including chickens, these resources are poorly integrated and reused, greatly limiting genetic research and breeding processes of the species.

**Results:**

Here, we constructed Galbase, an easily accessible repository that integrates public chicken multi-omics data from 928 re-sequenced genomes, 429 transcriptomes, 379 epigenomes, 15,275 QTL entries, and 7,526 associations. A total of 21.67 million SNPs, 2.71 million InDels, and 488,583 cis-regulatory elements were included. Galbase allows users to retrieve genomic variations in geographical maps, gene expression profiling in heatmaps, and epigenomic signals in peak patterns. It also provides modules for batch annotation of genes, regions, and loci based on multi-layered omics data. Additionally, a series of convenient tools, including the UCSC Genome Browser, WashU Epigenome Browser, BLAT, BLAST, and LiftOver, were also integrated to facilitate search, visualization, and analysis of sequence features.

**Conclusion:**

Galbase grants new opportunities to research communities to undertake in-depth functional genomic studies on chicken. All features of Galbase make it a useful resource to identify genetic variations responsible for chicken complex traits. Galbase is publicly available at http://animal.nwsuaf.edu.cn/ChickenVar.

**Supplementary Information:**

The online version contains supplementary material available at 10.1186/s12864-022-08598-2.

## Background

Technological advances and low sequencing costs have led to the generation and accumulation of a large amount of omics data, which are conducive to understand the genetic architecture of complex traits in farm animals and poultry. The availability of multi-omics data in large cohorts provides opportunities to systematically quantify the genetic control of DNA methylation, genetic and epigenetic regulations of gene expression, and their effects on complex traits. In addition to providing meat and eggs to consumers, chickens are widely used in developmental biology, medical research, and phenotypic evolution studies as a model organism. Modern chickens were domesticated from their wild ancestor, the red jungle fowl (RJF) [1]. Subsequently, chickens have been developed into different breeds exhibiting remarkable differences in morphology, behavior, physiology, and adaptation [2]. Up to now, most genetic studies on chickens were conducted on one type of omics data with a single analysis method, such as differential expression analysis [3], genome-wide association studies (GWAS) [4, 5], and selective sweep analysis [6, 7]. However, these studies only described the characteristics of data from one level and could not detect credible candidate variations and genes based on limited evidence. A few studies have attempted to reveal the genetic basis for complex traits of chickens using multi-omics datasets, such as for small body size of Yuanbao chicken [8], silky-feather [9], and skin pigmentation phenotype [10] of Silkie chicken, and blue eggshell phenotype of Dongxiang chicken [11]. These studies have narrowed the large candidate lists by screening the overlapping regions based on the evidence from different layers of multi-omics data and experimental verification, demonstrating the need for integrated system-level approaches for analyzing multi-omics data for complex traits. Therefore, developing a comprehensive muti-omics database can help users prioritize the candidate variations and genes, select credible genes for follow-up experimental verification, and apply them to chicken molecular breeding programs.

Currently, several chicken genomics and functional genomics databases have been developed. For instance, GEISHA is a repository of the metadata for genes expressed during the first-six-days of chicken embryo development identified by in situ hybridization analysis [12]. Chickspress is a database to collect gene expression of mRNAs, miRNAs, proteins, and peptides based on different tissue types at different developmental stages [13]. ChickenSD provides whole-genome SNPs from 863 re-sequenced chicken genomes [1]. Apart from these dedicated databases, some generic databases, including EBI/EVA [14], SNPchiMp v.3 [15], Expression Atlas [16], ECRbase [17], Animal QTLdb [18], and GWAS Atlas [19], have also collected different aspects of chicken data. Despite being very useful, these existing databases only focus on one specific omics area and provide a limited amount of data, making them unsuitable for inferring and studying complex traits.

To fill this gap, we developed Galbase, a multi-omics repository to integrate reference genomes, annotations, high-quality genetic variants, transcriptomes, histone modifications, open chromatin regions, variant-trait associations, and QTLs for chicken genetic research and breeding. We used a uniform pipeline to identify SNPs and InDels; evaluated the genetic diversity of each breed; introduced a tissue-specific index, tau, to help determine tissue expression patterns and tissue-specific genes; and integrated the WashU Epigenome Browser to visualize gene regulation patterns. With its rich annotations and functionalities, Galbase will serve as an important public resource to narrow the range of trait candidate genes and to mine for functional variations by selecting the intersections of evidence from different genomic levels.

## Construction and content

### Resequencing data alignment and variant calling

We integrated whole-genome sequencing data from a total of 928 chicken samples of different breeds, indigenous populations, and ecotypes based on previously published studies [1, 20, 21] [Date of data collection: April, 2021]. The data-set contained 778 domestic chickens (including 47 breeds) and 150 RJFs (including five RJF subspecies), totaling 7,279 Gb of sequencing data (Additional file 1: Table S1). We first trimmed low-quality reads and adapter sequences using Trimmomatic v0.36 [22]. Clean reads were then aligned against the GRCg6a chicken reference genome [GCF_000002315.6] using the default parameters of BWA-MEM v0.7.15 [23]. The BAM files were processed using Picard v2.1, including position sorting, sample merging, and marking duplicates and removal. The Genome Analysis Toolkit (GATK) v3.7 [24] HaplotypeCaller and GenotypeGVCFs algorithms were used to call SNPs and InDels according to previously published methods [25, 26]. We next filtered SNPs/InDels using the parameters “DP < TotalReadDepth/3 || DP > TotalReadDepth*3 || QD < 2.0 || QUAL < 30.0 || MQ < 40.0 || FS > 60.0 || SOR > 3.0 || ReadPosRankSum <  − 8.0 || MQRankSum < -12.5” / “DP < TotalReadDepth/3 || DP > TotalReadDepth*3 || QD < 2.0 || QUAL < 30.0 || MQ < 40.0 || FS > 200.0 || SOR > 10.0 || ReadPosRankSum <  − 20.0 || MQRankSum < -12.5”. We only retained the 1–30 bp indels. Finally, we downloaded a gff3 file for GRCg6a from NCBI and annotated these variations by using SnpEff v.4.3 [27]. The allele frequency of each chicken group and minor allele frequency (MAF) of all chickens were calculated with VCFtools [28] and PLINK [29], respectively.

### Genetic diversity and inbreeding coefficient

The nucleotide diversity of each group was estimated by VCFtools, using the parameters “–window-pi 50,000 –window-pi-step 25,000”. The ROH (Runs of homozygosity) of each group was calculated with PLINK parameters “–homozyg-window-snp 50 –homozyg-snp 50 –homozyg-kb 500 –homozyg-density 50 –homozyg-window-missing 5 –homozyg-window-threshold 0.05 –homozyg-window-het 3”. We then calculated genomic inbreeding coefficients (*F*_ROH_) by the formula: *F*_ROH_ = L_ROH_/L_total_ [30, 31]. To avoid the bias due to sample size variation, we reduced the sample size of each group down to five, following random sampling 10 times, then compared with the calculation results of the original samples.

### RNA-seq data processing

We downloaded 429 RNA-seq datasets covering 44 tissues of chicken (Additional file 2: Table S2) from NCBI Sequence Read Archive (SRA) [Date of data collection: March, 2022]. The raw data were pre-processed to filter low-quality reads and remove adaptor sequences by using Trimmomatic v0.36 [22]. Clean reads were then aligned against the chicken GRCg6a reference genome with STAR v2.5.1 [32], and unmapped reads were extracted to perform the second alignment by HISAT2 v2.0.3-beta [33] to improve their utilization [34]. Each bam file was then merged by the Picard tool (v2.1.1). The Transcripts Per Million (TPM) values were computed by StringTie v1.3.4 [35]. The tissue-specific index, tau [36], was calculated by an in-house python script. For some specific experimental designed groups derived from the same project, we performed differential expression analysis using DESeq2 [37].

### Epigenome data processing

All available chicken epigenomic data [Date of data collection: March, 2022] were downloaded from NCBI SRA, including histone ChIP-seq and ATAC-seq (Additional file 3: Table S3). Trim Galore was used to trim adaptor and low-quality bases, then cleaned reads were aligned against the chicken GRCg6a reference genome using Bowtie2 v2.2.8 [38]. Low-quality and multiple-mapping reads were removed by using samtools [39] with the option ‘‘-q 20’’. Reads mapping to mitochondrial DNA were also removed for subsequent analysis. The coverage of reads was calculated by the subroutine “bamcoverage” of deepTools [40]. MACS2 v.2.1.1 [41] was used to call peaks with the option “-q 0.05”. The enriched peaks were defined as potential regulatory regions. We first classified the epigenome data into different categories according to the experiment type, such as the H3K4me3 marked as active promoters, H3K27ac marked as active promoters and enhancers, H3K4me1 marked as enhancers and other distal regulatory elements, H3K27me3 marked as repressed transcription, CTCF marked to maintain genome 3D structure, and ATAC-seq marked as open chromatin regions. Next, we overlapped the enriched regions of repeated samples using the BEDtools intersect function, then merged the different tissues using the BEDtools merge function. The total number and total length of the set are used to define the potential regulatory elements of each classification. Finally, we combined all the categories to count the total number and length of regulatory elements in the whole chicken genome.

### Phenotypic information collection

We collected chicken phenotypic traits from Animal QTLdb [18], GWAS Atlas [19], and previously reported studies (Additional file 4: Table S4). For Animal QTLdb, we retained entries containing both chromosomal locations and trait names. For variant-trait associations, we collected data from GWAS Atlas and published literature resources. Since GWAS Atlas only collected 10 chicken publications, we re-searched literature in the NCBI PubMed database using “chicken” and “GWAS” as keywords [Date of data collection: April, 2022]. We manually curated 7,526 associations and 192 traits from 62 publications (Additional file 4: Table S4). We used the chicken GRCg6a reference genome as the reference physical map to convert all phenotypic data to this version using liftOver.

### Projection of GRCg6a genomic features to the GRCg7b reference genome

We performed pairwise alignment from GRCg7b [GCF_016699485.2] to GRCg6a by using minimap2, converted the results to liftOver chain files, and then configured an online coordinate conversion. In order to be compatible with the latest chicken reference genome (GRCg7b), we also transformed the coordinates of all muti-omics genomic features to the newer assembly by using the LiftOver tool [42] with the default parameters, which will allow users to retrieve multi-omics data through the two most recently assembled genome assemblies (GRCg6a and GRCg7b).

### Database implementation

Galbase was built in a LAMP (Linux + Apache + MySQL + PHP) environment, and implemented by a model-view controller (MVC) design pattern based on CodeIgniter framework. The web interfaces were written by PHP, HTML, CSS, JavaScript language, jQuery, Bootstrap, and the open-sourced ECharts framework [43]. The UCSC Genome Browser [42], WashU Epigenome Browser [44], BLAST [45], BLAT, and liftOver [42] functions were also built and adjusted to adapt to our database. We used in-house Perl scripts to process all multi-omics data and manage this large volume of information through MySQL database. The website has been tested in mainstream browsers such as Internet Explorer (version >  = 9), Firefox, Google Chrome, and Safari.

## Utility and discussion

### Omics data collection and statistics

**Fig. 1:**
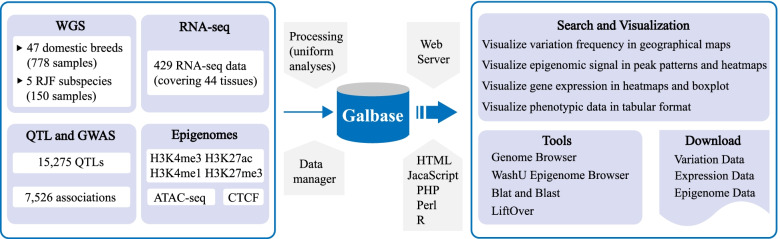
The database structure and processing pipeline of Galbase

Galbase collected the public chicken multi-omics data of 928 re-sequenced genomes (Additional file 1: Table S1), 429 transcriptomes (Additional file 2: Table S2), 379 epigenomes (Additional file 3: Table S3), 15,275 QTL entries (Data from Animal QTLdb), and 7,526 associations (Additional file 4: Table S4). After applying quality control and standardized data processing procedures, these datasets have been converted to usable table information in the MySQL database (Fig. 1).

**Fig. 2:**
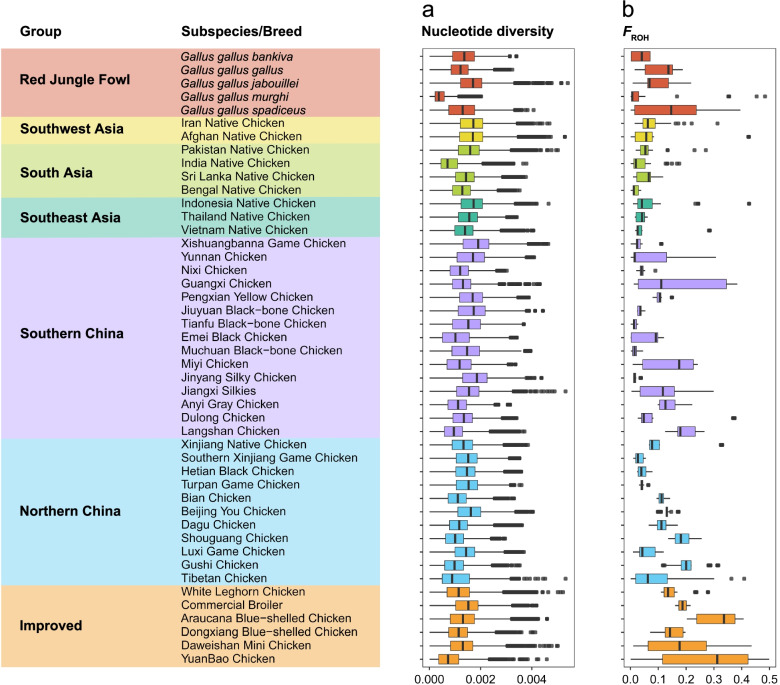
Statistics of population genomic diversity based on random sampling of each group (To avoid the bias due to sample size variation, we reduced the sample size of each group down to five, following random sampling for 10 times.). **a** Genome-wide distribution of nucleotide diversity in each chicken group. **b** Distribution of *F*_ROH_ estimate in each chicken group

The final list of variations included 21,672,487 SNPs and 2,708,244 InDels, which were annotated into 25 consequence types (Additional file 4: Table S5 and S6). We estimated π and *F*_ROH_ values to evaluate the genomic diversity of each breed (Fig. 2 and Additional file 4: Fig. S1). We found higher level of nucleotide diversity (Fig. 2a and Additional file 4: Fig. S1a) and relatively lower level of inbreeding coefficients (Fig. 2b and Additional file 4: Fig. S1b) in RJFs than domestic chickens, representing a higher level of genetic diversity in wild populations. In domestic chickens, breeds in Southwest Asia, South Asia, Southeast Asia, and Southern China had higher level of nucleotide diversity (Fig. 2a and Additional file 4: Fig. S1a) but lower level of inbreeding (Fig. 2b and Additional file 4: Fig. S1b) than those in Northern China, due possibly to more frequent gene flow between RJFs and sympatric domestic chickens [1]. Some highly selected breeds and native breeds, including White Leghorn, Commercial broilers, Araucana Blue-shelled chicken, Dongxiang Blue-shelled chicken, Daweishan Mini chicken, Yuanbao chicken, Guangxi chicken, Miyi chicken, Jiangxi Silkies, Anyi Gray chicken, Langshan chicken, Shouguang chicken, and Gushi chicken, showed higher levels of inbreeding (Fig. 2b and Additional file 4: Fig. S1b), indicating potential risks of their extinction following inbreeding depression and thus calling for conservation measures [31, 46].

The transcriptome data covers 44 tissues at different developmental stages. We added some valuable groups to provide information for chicken trait studies, such as high and low altitude samples, slow and fast-growing muscles, as well as normal and frizzle shaped feathers. We calculated TPM values and tissue specific index (tau) for 23,729 genes. The gene ontology (GO) analysis validated tissue-specific genes as being involved in the known tissue-relevant biological processes (Additional file 4: Table S7). We also performed differential expression analysis for each experimental designed group from the same project and provided a list of differentially expressed genes. To better interpret the changes in expression levels, we collected and included four histone modification marks (H3K4me3, H3K27ac, H3K4me1, and H3K27me3) and one transcription factor, CCCTC-binding factor (CTCF), based on ChIP-seq, as well as one open chromatin marker, based on ATAC-seq, to identify cis-regulatory elements. A total of 488,583 cis-regulatory elements were identified, accounting for 49.37% of the whole genome size (Additional file 4: Table S8).

The phenotypic data were collected from AnimalQTLdb, GWAS Atlas, and public literature resources (Additional file 4: Table S4). We mapped reported genes and positional information for all collected phenotypic data to the chicken GRCg6a genome. The data includes 609 different chicken traits which were divided into 15 major categories (Additional file 4: Table S9). We found that the reported traits were mainly concentrated in the “Growth Related Traits”, “Egg Related Traits”, “Exterior Features”, “Behavior Related Traits”, and “Feeding Related Traits” categories, which is consistent with the mainstream research on chickens.

### Database characteristics

Galbase comprises a data storage warehouse though MySQL, a user search engine by CodeIgniter, and a set of tools for analysis and visualization. We categorized the chicken multi-omics data into five main retrieval functionalities: (i) variation module; (ii) expression module; (iii) epigenomic module; (iv) phenotypic module; (v) batch annotation; and (vi) a series of useful tools. Each module has its own page, and features are linked to each other by gene symbols and chromosomal locations.

### Variation module

**Fig. 3:**
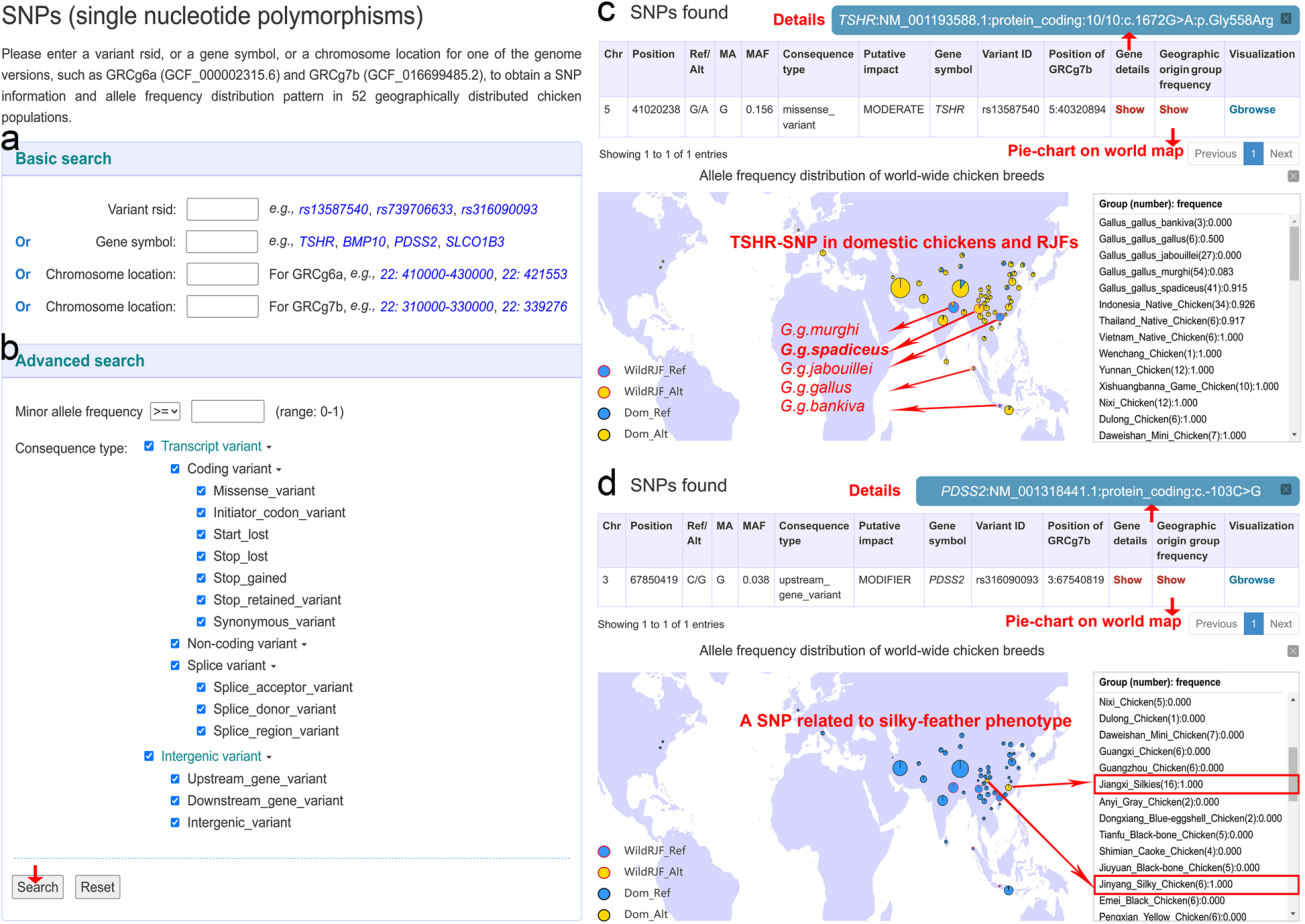
Features of Galbase variation module. **a** Basic search interface includes filtering of variation rsID, gene symbol, and chromosomal location of both GRCg6a and GRCg7b assemblies. **b** Advanced search interface includes filtering of minor allele frequency and consequence type. **c** An example shows the chr5:41,020,238 locus at the *TSHR* gene and its allele frequency distribution of five RJF subspecies and 47 domestic chicken breeds. **d** An example shows the chr3:67,850,419 locus at the *PDSS2* gene and its allele frequency distribution. The maps were created by using ECharts (https://github.com/apache/echarts) [43], an open-sourced and web-based framework based on JavaScript

The “Variation module” was designed to dynamically retrieve relevant SNP and InDel information in a tabular format or in a genome browser interface. Simply by specifying a variant rsID, a gene symbol, or a chromosomal location (Fig. 3a), users can easily obtain the results of a query for all annotated variation information, including chromosome, position, reference/alternative alleles, MAF, consequence type, variant ID, and allele frequency (Fig. 3c and 3d). Gbrowse (integrated from UCSC Genome Browser, here we named it “Gbrowse”) was linked to this page to help view other sequence features (Fig. 3c and 3d). More filtering parameters can be set to obtain those variations that fall in different gene bodies or non-protein-coding fraction (Fig. 3b). Moreover, a geographical map showing the allele frequency of five RJF subspecies and 47 chicken breeds can also be displayed, accompanied by each search (Fig. 3c and 3d). This function can help users to identify the breed- or trait-associated variants.

### Expression module

**Fig. 4:**
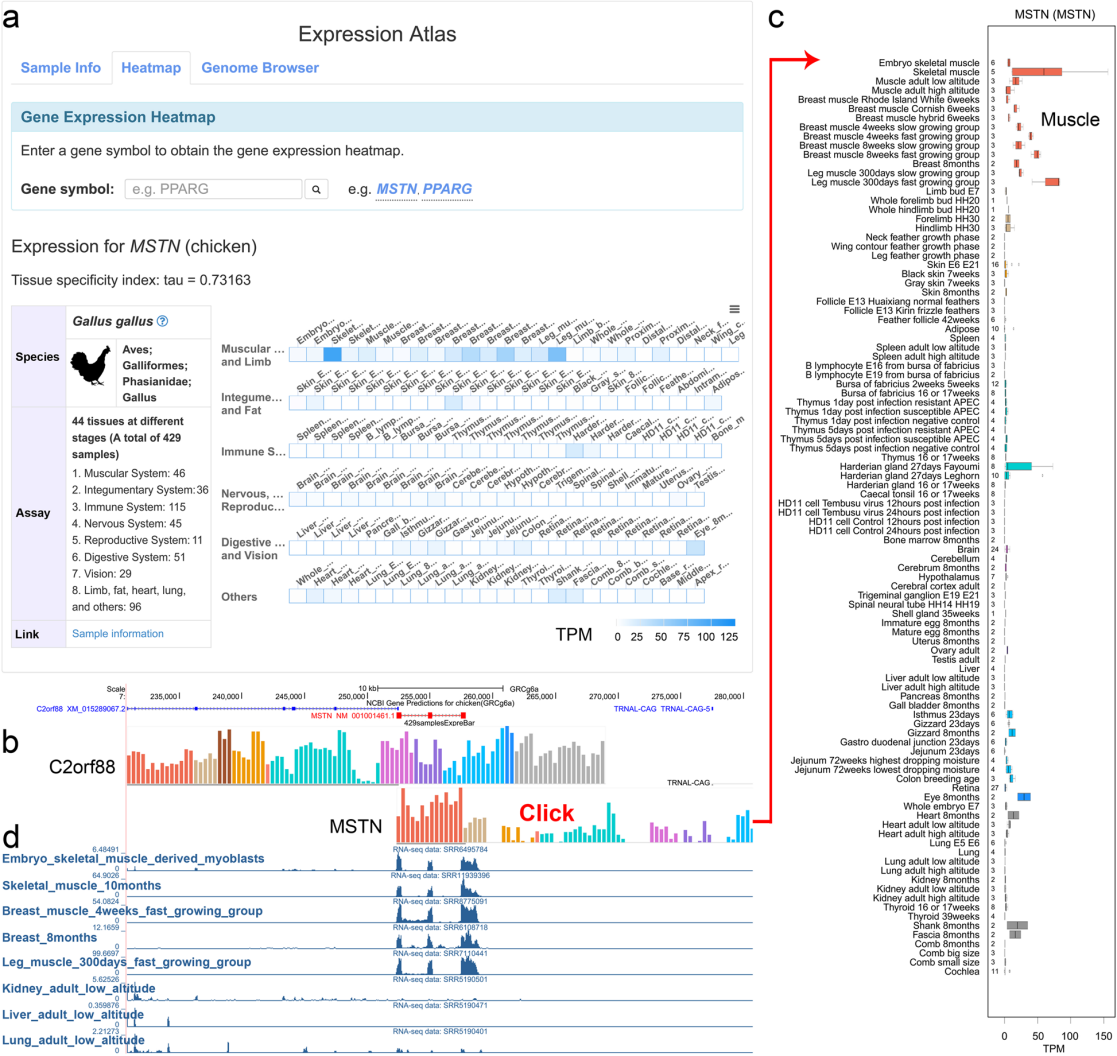
Features of Galbase expression module. **a** Screenshots of gene expression function and the result for one example. (The chicken illustration is processed from a chicken photo taken by Weiwei Fu, one of the authors of this article.) **b** Display of transcriptome expression histogram in Genome Browser. **c** A boxplot showing the range of expression levels across all chicken samples by clicking the gene symbol in Fig. 4b. Different colors represent different organ systems. **d** Display of the coverage depth of transcriptome reads in Genome Browser

The “Expression module” displays gene expression profiles in three ways. The first displays the gene expression matrix by heatmap and also incorporates a tau value (Fig. 4a), which enables users to easily distinguish gene expression patterns and tissues with specific or high abundance expression. The other two ways display RNA-seq data in Gbrowse. The “expreBar” track (Fig. 4b) can be linked to a more detailed boxplot display page (Fig. 4c) by clicking one gene symbol, where users can view the expression levels of all samples (Fig. 4c). The “RNAseqReadsCoverage” track, displays normalized read coverage depth by converting BAM files to 1 × sequencing depth, so that users can compare the expression levels between different samples (Fig. 4d). We also performed differential expression analysis by DESeq2 [37] for some specific experimental designed groups, and provide upregulated and downregulated gene lists. Users can download expression matrices and differential gene lists in a CSV format or plain-text files for further analysis.

### Epigenomics module

**Fig. 5:**
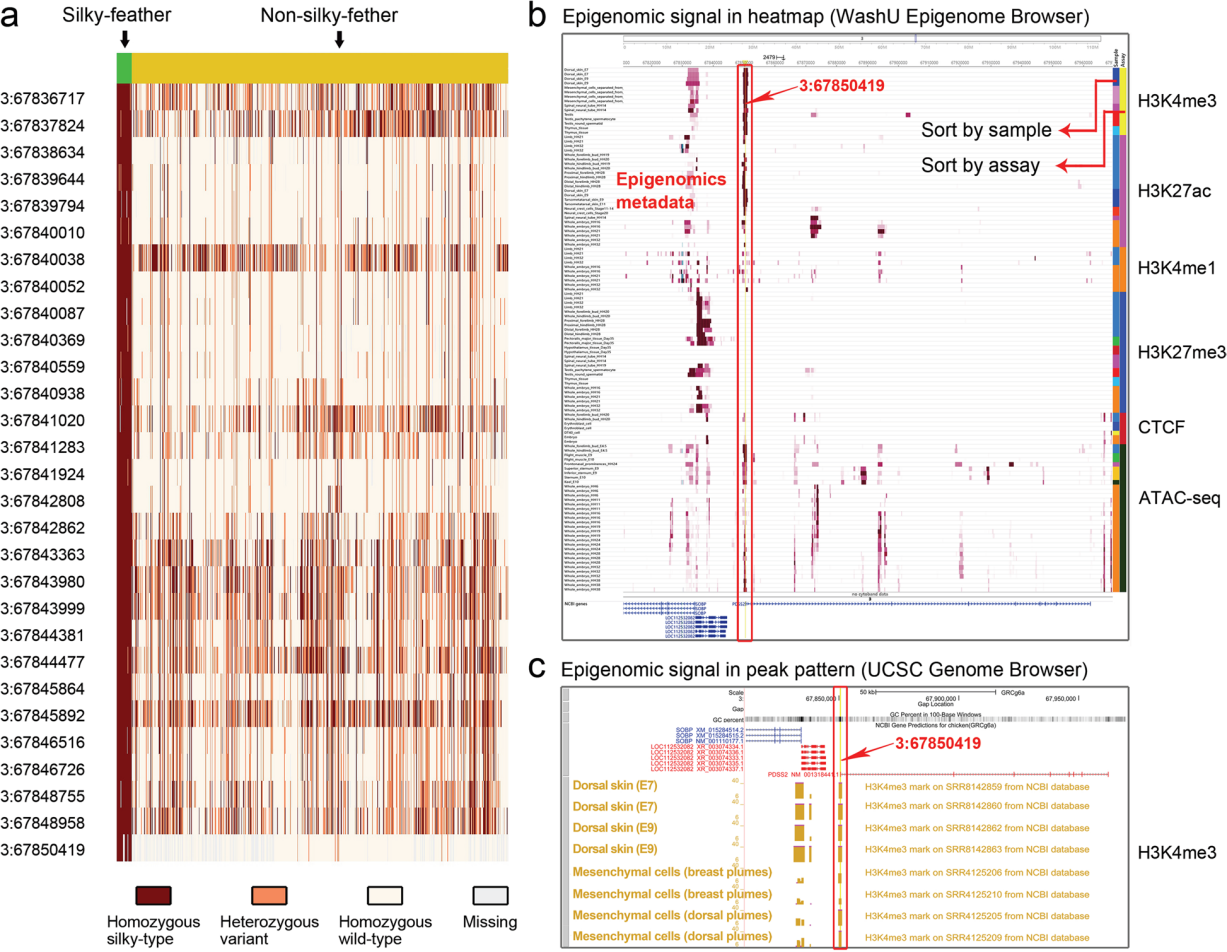
Presentation of multi-omics data for silky-feather trait. **a** Genotype pattern of the silky-feather fixed region (chr3:67,832,192–67,850,863). Only the chr3:67,850,419 locus conforms to a single gene recessive inheritance mode. **b** Display of epigenomics metadata in local WashU Epigenome Browser. **c** Display of epigenomics peaks and read coverage in local UCSC Genome Browser

Cis-regulatory elements can help elucidate the causes of complex traits and altered expression levels. Galbase encompasses data relating to four histone modifications: H3K4me3 (active promoters), H3K27ac (active promoters and enhancers), H3K4me1 (enhancers and other distal regulatory elements), H3K27me3 (repressed transcription); one transcription factor, CCCTC-binding factor (CTCF), contributing to 3D genome organization; and one open chromatin marker based on ATAC-seq to identify regulatory regions. The epigenomics metadata, including sample information, experiment type, and epigenetic mark, can be displayed by heatmap views or typical ‘wiggle’ views in WashU Epigenome Browser (Fig. 5b). The epigenomics data can also be retrieved by a table browser. Both regulatory peaks and read coverage (normalized 1 × sequencing depth) can be visualized in Gbrowse (Fig. 5c). Tracks can be sorted, organized, dragged, and printed in a PDF format based on user preferences.

### Phenotypic module

The “Phenotypic module” contains 15,275 QTL entries and 7,526 variant-trait associations manually curated from AnimalQTLdb [18], GWAS Atlas [19], and literature resources. To unify different chicken traits, we divided the trait entities into 15 major categories using the standard classification of chicken QTLdb (Additional file 4: Table S9). We provided various ways to browse and retrieve the phenotypic data: search by gene symbol, search by variant rsid, find QTLs or associations by genome location, and find associated genes by a trait name or a keyword. In order to be compatible with the latest version of the chicken reference genome, we transformed the coordinates to the newest genome assembly by using the LiftOver tool [42] with the default parameters.

### Batch annotation

This module was designed on the home page to realize full database retrieval. We integrated all aforementioned multi-omics data to annotate genes, genomic regions, and loci in batches, which can improve the accuracy and reliability of screening, and help users to better analyze and judge the function of genes. Users can enter a candidate list of genes or positions to view all available datasets: (i) SNPs; (ii) InDels; (iii) Epigenomics; (iv) Expression; (v) QTL; (vi) GWAS; (vii) Gene Ontology; and (viii) KEGG pathway. The search results are presented in a tabular format and can be downloaded in a CSV format for downstream analyses.

### A series of useful tools

In order to facilitate the usage of the database and to compensate for the lack of tools in the latest chicken GRCg7b reference genome, we have introduced and built several commonly used tools. The currently available tools included two genome browsers (the UCSC Genome Browser [42] and WashU Epigenome Browser [44]), a BLAST server [45], a BLAT server, and a liftOver tool [42]. Users can use the UCSC Genome Browser to visualize the multi-omics data in a global view. Currently, 1196 tracks for the GRCg6a assembly and 388 tracks for GRCg7b assembly have been released. Users can view SNPs, InDels, gene expression, epigenomics signal, and phenotypic data by searching for a gene symbol or a genomic region. The WashU Epigenome Browser was designed to display epigenomic data specially and we configured the epigenomic data to the Browser. The BLAST and BLAT servers target GRCg6a and GRCg7b genome assemblies, which allows researchers to perform sequence alignment, locate the position of the sequence on the genome, and infer the sequence function. The liftOver tool can offer an online coordinate conversion from GRCg6a to GRCg7b and chain files can be downloaded to support the liftOver server version.

### Example applications and discussion

Establishing a systematic multi-omics database is critical to streamline all mega-datasets to provide an easy access for different users in the field of animal genetics and breeding, however, such databases are very limited in domestic animals. Here, we use chicken as a paradigm for archive, analysis, and visualization of muti-omics data on genome-wide SNPs, InDels, expression, epigenomics, GWAS, and QTL. Compared with other specialized databases, including GEISHA [12], Chickspress [13], and ChickenSD [1], Galbase excels in the following two aspects:

First, Galbase provides variants and their allele frequency for both wild and domestic chickens in nearly 1000 genomes, which can help investigation of the population history of chickens. For instance, a previous study reported a missense mutation in the *TSHR* gene (GRCg6a; chr5:41,020,238 G/A; TSHR-Gly558Arg) to be a domestication locus since it was nearly fixed in all domestic chickens [2]. However, subsequent studies found that the frequency of TSHR-558Arg mutation in European archaeological chickens sharply increased only in the last 1000 years [47], while this allele also had a very high frequency in the ancestor of domestic chickens [1], *Gallus gallus spadiceus*, suggesting this mutation may not be a domestication locus following the complex domestication history of all chickens. By querying our database, users can easily obtain the frequency distribution pattern of domestic chickens and RJFs (Fig. 3c). This intuitive display of geographic allele frequency can help quickly verify hypothesized population history.

Secondly, Galbase provides multi-omics data to help comprehensively judge the potential causal variation of chicken complex traits. For instance, the silky-feather phenotype is controlled by a single recessive gene [9, 48]. We calculated the *F*_ST_ values of silky-feather and non-silky-feather groups though the vcf file that was downloaded from our database. We selected highly differentiated loci (*F*_ST_ > 0.4) (Additional file 4: Table S10) in the previously reported fine mapping interval of the silky-feather phenotype (GRCg6a; chr3:67,832,192–67,850,863) [9], and presented its genotype patterns (Fig. 5a). We found that only the chr3:67,850,419 locus conformed to the single gene recessive inheritance mode, that is, the homozygous silky-type did not exist in the non-silky-feather group. By querying the epigenomics data in our database, we found that chr3:67,850,419 was located in a region showing strongly-enriched signals for H3K4me3, H3K27ac, and ATAC-seq (Fig. 5b and 5c), which was consistent with the published experimental results showing that the chr3:67,850,419 locus leads to the silky-feather phenotype by affecting promoter activity [9]. This exemplifies the use of multi-omics resources obtained from Galbase to reveal complex traits and reduce the verification work of downstream experiments. In addition, Galbase provides a variety of downloadable forms of expression data. Users can easily screen tissue-specific genes according to tau index or filter differentially expressed genes according to the result of DEseq2, which makes it more convenient to investigate tissue traits.

### Data management plan and future update

We will continue to incorporate newly released chicken multi-omics data, and provide dedicated tools required to explore and visualize these data. In order to connect and integrate these external resources more quickly, we will develop an automatic interface to download daily published data, upload it to the supercomputer platform for quality control, processing and analysis when the sample size gets larger than 100 individuals, and finally process the offline data into the website format. For the analysis of re-sequenced genomes, which require a lot of computational resources, we will maintain a major update every year. Our plan for the next phase is to use deep learning algorithms to integrate multi-omics data, which will provide a comprehensive insight from genotype to phenotype, so as to better evaluate and mine heterogeneous multi-omics information.

## Conclusions

We present a comprehensive chicken multi-omics database, named Galbase, for the identification of credible candidate genes and loci from different omics layers. To make the data easily accessible, and the usage of the information more effective and flexible, Galbase offers several convenient modules and tools to retrieve, present, and analyze abundant genetic variants, transcriptomic, epigenomic, and phenotypic data, which can help uncover the biomechanisms behind complex traits. Galbase provides the largest integrated chicken data repository to date and will help provide new functional insights from genomic data thus offering great promise for the chicken research community.

## Supplementary Information

**Additional file 1:** 

**Additional file 2:** 

**Additional file 3:** 

**Additional file 4:** 

## Data Availability

Galbase is freely available at http://animal.nwsuaf.edu.cn/ChickenVar. All data used in this study are available from NCBI SRA and PubMed database. The accession numbers analyzed in this study are listed in Additional file 1: Table S1, Additional file 2: Table S2, Additional file 3: Table S3, and Additional file 4: Table S4. And two chicken genome assemblies can be accessed on NCBI Assembly database via the accession numbers: GRCg6a (GCF_000002315.6) and GRCg7b (GCF_016699485.2).

## Abbreviations

QTL: Quantitative Trait Locus
RJF: Red jungle fowl
GWAS: Genome-wide association studies
MAF: Minor allele frequency
SRA: Sequence Read Archive
TPM: Transcripts Per Million
MVC: Model-view controller
Tau: Tissue specific index
CTCF: CCCTC-binding factor
