# Supplementary material for: Galbase: a comprehensive repository for integrating chicken multi-omics data
Source: BMC Genomics. 2022 May 12;23:364. doi: 10.1186/s12864-022-08598-2 (PMC9097087; doi:10.1186/s12864-022-08598-2)
Supplement: Supplementary file 4 — Additional file 4: [file 12864_2022_8598_MOESM4_ESM.docx]

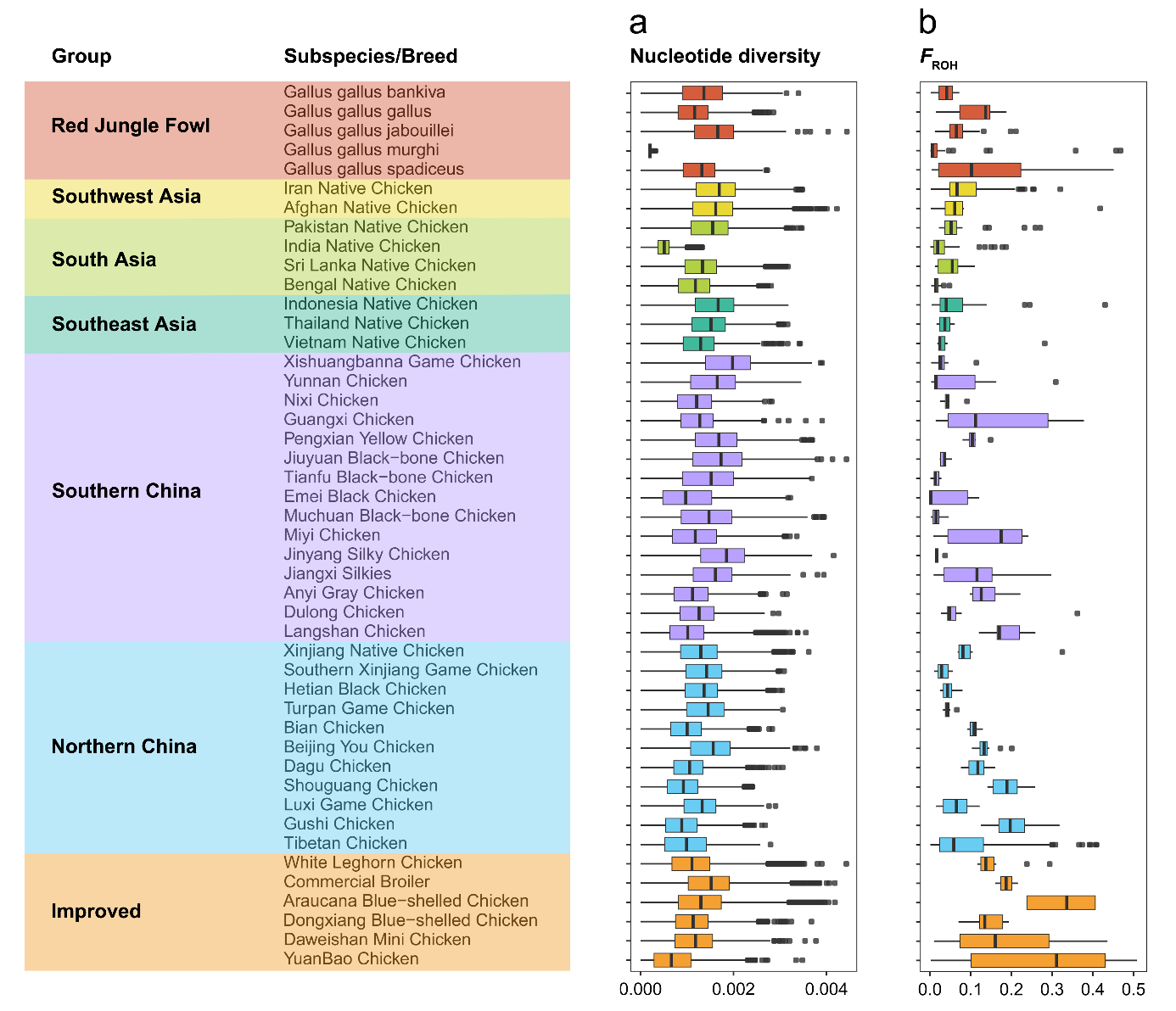


**Fig. S1** Statistics of population genomic diversity for five subspecies of red jungle fowl and domestic chicken breeds. **a** Genome-wide distribution of nucleotide diversity in each group. **b** Distributions of *F*_ROH_ estimate in each chicken group.

**Table S4** **Summary information of 62 publications about GWAS used for extracting associations.**

| **PubMed ID** | **Publications** |
| --- | --- |
| 34855995 | New insights into genetics underlying of plumage color |
| 34904261 | Deletion in KRT75L4 linked to frizzle feather in Xiushui Yellow Chickens |
| 34991478 | Genome-wide association study identifies SNPs for growth performance and serum indicators in Valgus-varus deformity broilers (Gallus gallus) using ddGBS sequencing |
| 35052342 | Large-Scale Whole Genome Sequencing Study Reveals Genetic Architecture and Key Variants for Breast Muscle Weight in Native Chickens |
| 35218583 | An association of CEP78, MEF2C, VPS13A and ARRDC3 genes with survivability to heat stress in an F 2 chicken population |
| 35225379 | Genetic parameters estimation and genome-wide association studies for internal organ traits in an F 2 chicken population |
| 35299951 | Genome-Wide Association Studies Provide Insight Into the Genetic Determination for Hyperpigmentation of the Visceral Peritoneum in Broilers |
| 33398896 | Genome-wide association study of body size traits in Wenshang Barred chickens based on the specific-locus amplified fragment sequencing technology |
| 33516007 | Genome-wide association studies for mottled eggs in chickens using a high-density single-nucleotide polymorphism array |
| 33549052 | Identification of QTL regions and candidate genes for growth and feed efficiency in broilers |
| 33633287 | Exploring the genetic architecture of feed efficiency traits in chickens |
| 33995468 | Genomic Regions Related to White/Black Tail Feather Color in Dwarf Chickens Identified Using a Genome-Wide Association Study |
| 34376144 | Identification of candidate genomic regions for chicken egg number traits based on genome-wide association study |
| 34408769 | Genome-Wide Association Study Using Whole-Genome Sequencing Identifies a Genomic Region on Chromosome 6 Associated With Comb Traits in Nandan-Yao Chicken |
| 34440344 | Genome-Wide Association Study Identifies 12 Loci Associated with Body Weight at Age 8 Weeks in Korean Native Chickens |
| 34496773 | A genome-wide association analysis for body weight at 35 days measured on 137,343 broiler chickens |
| 34727889 | Genetic variations for the eggshell crystal structure revealed by genome-wide association study in chickens |
| 34745207 | Genome-Wide Association Study of Growth Performance and Immune Response to Newcastle Disease Virus of Indigenous Chicken in Rwanda |
| 24333371 | A genome-wide association study identifies major loci affecting the immune response against infectious bronchitis virus in chicken |
| 25260525 | A genome-wide association study identifies novel single nucleotide polymorphisms associated with dermal shank pigmentation in chickens |
| 26365057 | Genome-wide association study of growth traits in Jinghai Yellow chicken hens using SLAF-seq technology |
| 26614681 | Genome-wide association study of 8 carcass traits in Jinghai Yellow chickens using specific-locus amplified fragment sequencing technology |
| 26853217 | Genomewide association study of immune traits in chicken F2 resource population |
| 26859147 | Parallel Evolution of Polydactyly Traits in Chinese and European Chickens |
| 27166871 | Genome-wide association study reveals novel variants for growth and egg traits in Dongxiang blue-shelled and White Leghorn chickens |
| 27427764 | Genome-Wide Association Studies for Comb Traits in Chickens |
| 27456605 | Genetic architecture dissection by genome-wide association analysis reveals avian eggshell ultrastructure traits |
| 27485826 | Genome-wide association study of aggressive behaviour in chicken |
| 27506765 | Combination analysis of genome-wide association and transcriptome sequencing of residual feed intake in quality chickens |
| 27687164 | Genome-wide association studies of immune, disease and production traits in indigenous chicken ecotypes |
| 28158968 | A genome-wide association study in a large F2-cross of laying hens reveals novel genomic regions associated with feather pecking and aggressive pecking behavior |
| 28235410 | TCF21 is related to testis growth and development in broiler chickens |
| 28427323 | Detection of QTL for traits related to adaptation to sub-optimal climatic conditions in chickens |
| 28877683 | Genome-wide association study reveals putative role of gga-miR-15a in controlling feed conversion ratio in layer chickens |
| 29281706 | A genome-wide study to identify genes responsible for oviduct development in chickens |
| 29554873 | Mapping QTL for white striping in relation to breast muscle yield and meat quality traits in broiler chickens |
| 29672870 | Genome-wide association study confirms that the chromosome Z harbours a region responsible for rumplessness in Hongshan chickens |
| 29755503 | Genome-Wide Association Analysis of Age-Dependent Egg Weights in Chickens |
| 29936586 | Genome-wide association study on chicken carcass traits using sequence data imputed from SNP array |
| 29946990 | Genome-wide association study of body weight in Wenshang Barred chicken based on the SLAF-seq technology |
| 29954329 | Single SNP- and pathway-based genome-wide association studies for beak deformity in chickens using high-density 600K SNP arrays |
| 30381748 | Genome wide association study on feed conversion ratio using imputed sequence data in chickens |
| 30673708 | Genome-wide association study and a post replication analysis revealed a promising genomic region and candidate genes for chicken eggshell blueness |
| 30696883 | Genome wide association study of body weight and feed efficiency traits in a commercial broiler chicken population, a re-visitation |
| 30947682 | Genome-wide association studies targeting the yield of extraembryonic fluid and production traits in Russian White chickens |
| 31017703 | Genome-wide association study identified genes in the response to Salmonella pullorum infection in chickens |
| 31117270 | Genome-Wide Association Study of H/L Traits in Chicken |
| 31235723 | Discovery and characterization of functional modules associated with body weight in broilers |
| 31316551 | Genomic Analysis Reveals Pleiotropic Alleles at EDN3 and BMP7 Involved in Chicken Comb Color and Egg Production |
| 31319636 | Genetic Analyses of Tanzanian Local Chicken Ecotypes Challenged with Newcastle Disease Virus |
| 31412760 | Genome-wide association analysis of egg production performance in chickens across the whole laying period |
| 31533607 | A genome-wide association study explores the genetic determinism of host resistance to Salmonella pullorum infection in chickens |
| 31541310 | A Novel Model to Explain Extreme Feather Pecking Behavior in Laying Hens |
| 31656738 | Genetic architecture related to contour feathers density in an F 2 resource population via a genome-wide association study |
| 32183495 | Genome-wide Analyses Identifies Known and New Markers Responsible of Chicken Plumage Color |
| 32318090 | New Insights From Imputed Whole-Genome Sequence-Based Genome-Wide Association Analysis and Transcriptome Analysis: The Genetic Mechanisms Underlying Residual Feed Intake in Chickens |
| 32366026 | Genome-Wide Association Study of Muscle Glycogen in Jingxing Yellow Chicken |
| 32849779 | Genetic Basis of Response of Ghanaian Local Chickens to Infection With a Lentogenic Newcastle Disease Virus |
| 32849807 | The Genetic Architecture of the Chickens Dropping Moisture by Genetic Parameter Estimation and Genome-Wide Association |
| 32867375 | Genome-Wide Association Study and Pathway Analysis for Heterophil/Lymphocyte (H/L) Ratio in Chicken |
| 32989280 | Genome-wide association study reveals the genetic determinism of growth traits in a Gushi-Anka F 2 chicken population |
| 33004014 | Meta-analyses of genome wide association studies in lines of laying hens divergently selected for feather pecking using imputed sequence level genotypes |

**Table S5 Consequence type for identified SNPs**

| **Consequence Type** | **SNP Count** |
| --- | --- |
| intron_variant | 10343738 |
| intergenic_variant | 6411563 |
| upstream_gene_variant | 2156365 |
| downstream_gene_variant | 1395746 |
| 3_prime_UTR_variant | 492344 |
| synonymous_variant | 275419 |
| missense_variant | 176985 |
| non_coding_transcript_exon_variant | 171234 |
| 5_prime_UTR_variant | 153296 |
| splice_region_variant | 52433 |
| 5_prime_UTR_premature_start_codon_gain_variant | 37992 |
| stop_gained | 1924 |
| splice_donor_variant | 1596 |
| splice_acceptor_variant | 1135 |
| start_lost | 313 |
| stop_lost | 197 |
| stop_retained_variant | 180 |
| initiator_codon_variant | 27 |

**Table S6 Consequence type for identified InDels**

| **Consequence Type** | **InDel Count** |
| --- | --- |
| intron_variant | 1346802 |
| intergenic_variant | 776271 |
| upstream_gene_variant | 276807 |
| downstream_gene_variant | 185774 |
| 3_prime_UTR_variant | 64208 |
| 5_prime_UTR_variant | 22452 |
| non_coding_transcript_exon_variant | 18988 |
| frameshift_variant | 7113 |
| splice_region_variant | 5208 |
| disruptive_inframe_deletion | 1872 |
| conservative_inframe_deletion | 994 |
| splice_donor_variant | 467 |
| disruptive_inframe_insertion | 425 |
| conservative_inframe_insertion | 420 |
| splice_acceptor_variant | 371 |
| stop_gained | 39 |
| bidirectional_gene_fusion | 12 |
| start_lost | 9 |
| stop_lost | 9 |
| gene_fusion | 3 |

**Table S7 Gene Ontology (GO) enrichment analysis of tissue-specific expressed genes** (Genes with tau > 0.8 are defined as tissue-specifically expressed)

| **GO term ID** | **GO term** | **Corrected *P*-value** | **Gene count** |
| --- | --- | --- | --- |
| GO:0050877 | nervous system process | 8.10E-11 | 40 |
| GO:0007600 | sensory perception | 1.51E-10 | 29 |
| GO:0007601 | visual perception | 3.33E-10 | 21 |
| GO:0050953 | sensory perception of light stimulus | 3.33E-10 | 21 |
| GO:0003008 | system process | 3.33E-10 | 52 |
| GO:0043010 | camera-type eye development | 4.74E-03 | 17 |
| GO:0006357 | regulation of transcription by RNA polymerase II | 7.74E-03 | 57 |
| GO:0051606 | detection of stimulus | 7.74E-03 | 10 |
| GO:0001654 | eye development | 8.90E-03 | 18 |
| GO:0150063 | visual system development | 8.90E-03 | 18 |
| GO:0042391 | regulation of membrane potential | 8.90E-03 | 13 |
| GO:0006366 | transcription by RNA polymerase II | 8.90E-03 | 57 |
| GO:0048880 | sensory system development | 8.90E-03 | 18 |
| GO:0050906 | detection of stimulus involved in sensory perception | 8.90E-03 | 7 |
| GO:0014070 | response to organic cyclic compound | 1.04E-02 | 18 |
| GO:0060078 | regulation of postsynaptic membrane potential | 1.65E-02 | 9 |
| GO:0007423 | sensory organ development | 1.90E-02 | 21 |
| GO:0007389 | pattern specification process | 2.98E-02 | 18 |
| GO:0009566 | fertilization | 3.51E-02 | 6 |
| GO:0002088 | lens development in camera-type eye | 3.51E-02 | 8 |
| GO:0005576 | extracellular region | 2.27E-07 | 70 |
| GO:0005615 | extracellular space | 1.07E-04 | 49 |
| GO:0097730 | non-motile cilium | 1.16E-03 | 9 |
| GO:0001750 | photoreceptor outer segment | 1.62E-03 | 8 |
| GO:0097731 | 9+0 non-motile cilium | 1.62E-03 | 8 |
| GO:0097733 | photoreceptor cell cilium | 1.62E-03 | 8 |
| GO:0045211 | postsynaptic membrane | 9.63E-03 | 13 |
| GO:0030312 | external encapsulating structure | 1.01E-02 | 18 |
| GO:0031012 | extracellular matrix | 1.01E-02 | 18 |
| GO:0031226 | intrinsic component of plasma membrane | 1.20E-02 | 27 |
| GO:0097060 | synaptic membrane | 1.58E-02 | 13 |
| GO:0032993 | protein-DNA complex | 1.85E-02 | 11 |
| GO:0030141 | secretory granule | 1.93E-02 | 9 |
| GO:0005887 | integral component of plasma membrane | 1.93E-02 | 25 |
| GO:0034702 | ion channel complex | 2.26E-02 | 8 |
| GO:0005882 | intermediate filament | 2.80E-02 | 7 |
| GO:0045111 | intermediate filament cytoskeleton | 3.68E-02 | 7 |
| GO:0000981 | DNA-binding transcription factor activity, RNA polymerase II-specific | 1.03E-06 | 51 |
| GO:0003700 | DNA-binding transcription factor activity | 1.11E-06 | 52 |
| GO:0000977 | RNA polymerase II transcription regulatory region sequence-specific DNA binding | 1.84E-06 | 49 |
| GO:0003690 | double-stranded DNA binding | 3.74E-06 | 54 |
| GO:1990837 | sequence-specific double-stranded DNA binding | 3.74E-06 | 51 |
| GO:0043565 | sequence-specific DNA binding | 1.08E-05 | 52 |
| GO:0000976 | transcription cis-regulatory region binding | 1.08E-05 | 49 |
| GO:0001067 | transcription regulatory region nucleic acid binding | 1.08E-05 | 49 |
| GO:0030594 | neurotransmitter receptor activity | 1.72E-04 | 12 |
| GO:0005230 | extracellular ligand-gated ion channel activity | 2.00E-04 | 11 |
| GO:0140110 | transcription regulator activity | 2.00E-04 | 52 |
| GO:0005212 | structural constituent of eye lens | 2.00E-04 | 8 |
| GO:0000978 | RNA polymerase II cis-regulatory region sequence-specific DNA binding | 6.84E-04 | 38 |
| GO:0022824 | transmitter-gated ion channel activity | 6.90E-04 | 10 |
| GO:0022835 | transmitter-gated channel activity | 6.90E-04 | 10 |
| GO:0015276 | ligand-gated ion channel activity | 8.54E-04 | 12 |
| GO:0022834 | ligand-gated channel activity | 8.54E-04 | 12 |
| GO:0000987 | cis-regulatory region sequence-specific DNA binding | 8.54E-04 | 38 |
| GO:0098960 | postsynaptic neurotransmitter receptor activity | 1.42E-03 | 10 |
| GO:0005179 | hormone activity | 1.57E-03 | 12 |
| GO:0099529 | neurotransmitter receptor activity involved in regulation of postsynaptic membrane potential | 1.99E-03 | 9 |
| GO:1904315 | transmitter-gated ion channel activity involved in regulation of postsynaptic membrane potential | 1.99E-03 | 9 |
| GO:0022836 | gated channel activity | 2.83E-03 | 15 |
| GO:0015267 | channel activity | 4.00E-03 | 18 |
| GO:0022803 | passive transmembrane transporter activity | 4.00E-03 | 18 |
| GO:0004930 | G protein-coupled receptor activity | 7.33E-03 | 13 |
| GO:0003677 | DNA binding | 8.51E-03 | 62 |
| GO:0005198 | structural molecule activity | 8.83E-03 | 23 |
| GO:0048018 | receptor ligand activity | 8.83E-03 | 23 |
| GO:0030546 | signaling receptor activator activity | 9.67E-03 | 23 |
| GO:0004888 | transmembrane signaling receptor activity | 9.67E-03 | 26 |
| GO:0030545 | signaling receptor regulator activity | 1.06E-02 | 23 |
| GO:0031731 | CCR6 chemokine receptor binding | 1.43E-02 | 6 |
| GO:0005216 | ion channel activity | 1.95E-02 | 15 |
| GO:0001664 | G protein-coupled receptor binding | 2.01E-02 | 11 |
| GO:0048020 | CCR chemokine receptor binding | 2.06E-02 | 6 |
| GO:0005126 | cytokine receptor binding | 2.80E-02 | 11 |
| GO:0005231 | excitatory extracellular ligand-gated ion channel activity | 2.80E-02 | 6 |
| GO:0022848 | acetylcholine-gated cation-selective channel activity | 2.80E-02 | 6 |
| GO:0042379 | chemokine receptor binding | 2.80E-02 | 6 |
| GO:0038023 | signaling receptor activity | 3.67E-02 | 28 |
| GO:0060089 | molecular transducer activity | 3.67E-02 | 28 |

**Table S8 The data statistics of cis-regulatory elements**

| **Experiment** | **Number** | **Length** |
| --- | --- | --- |
| ATAC-seq | 512,240 | 314,730,940 |
| H3K4me3 | 121,956 | 94,461,003 |
| H3K27ac | 193,467 | 243,250,975 |
| H3K4me1 | 204,220 | 237,460,340 |
| H3K27me3 | 90,958 | 93,319,939 |
| CTCF | 108,620 | 48,860,705 |
| Merge all | 488,583 | 525,987,516 |

Genome proportion = Merge all (length)/Genome size= 49.37%

**Table S9 Phenotypic data classification and statistics**

| **Category** | **Number (traits)** |
| --- | --- |
| Growth Related Traits | 7315 |
| Egg Related Traits | 5214 |
| Exterior Features | 2643 |
| Behavior Related Traits | 1538 |
| Feeding Related Traits | 1527 |
| Pigmentation | 923 |
| Disease Susceptibility Traits | 940 |
| Reproduction Traits | 719 |
| Fat Related Traits | 477 |
| Meat Quality Traits | 445 |
| Blood Parameters Traits | 360 |
| Health-Other | 214 |
| Other | 201 |
| Production-Other | 158 |
| Digestive System | 127 |

**Table S10 Mutations with *F*_ST_ > 0.4 in silky-feather haplotype**

Note: Homozygous wild-type = Hom wild; Heterozygous variant = Het; Homozygous silky-type = Hom silk**y**

| **Position** | ***F*_ST_** | **ALT/REF** | **Gene** | **Silky-feather** | | | **Non-silky-feather** | | |
| --- | --- | --- | --- | --- | --- | --- | --- | --- | --- |
|  |  |  |  | **Hom**  **wild** | **Het** | **Hom**  **silky** | **Hom**  **wild** | **Het** | **Hom**  **silky** |
| 3:67836717 | 0.50 | C/T | SOBP | 0 | 1 | 25 | 371 | 223 | 119 |
| 3:67837824 | 0.45 | A/G | SOBP | 0 | 1 | 25 | 329 | 208 | 151 |
| 3:67838634 | 0.89 | C/T | SOBP | 0 | 0 | 26 | 629 | 48 | 17 |
| 3:67839644 | 0.86 | C/A | LOC112532082 | 0 | 2 | 24 | 638 | 72 | 14 |
| 3:67839794 | 0.88 | A/T | LOC112532082 | 0 | 0 | 24 | 651 | 64 | 15 |
| 3:67840010 | 0.84 | C/T | LOC112532082 | 0 | 2 | 24 | 624 | 84 | 19 |
| 3:67840038 | 0.45 | A/C | LOC112532082 | 0 | 0 | 26 | 323 | 237 | 152 |
| 3:67840052 | 0.85 | G/A | LOC112532082 | 0 | 0 | 26 | 622 | 87 | 17 |
| 3:67840087 | 0.84 | G/A | LOC112532082 | 0 | 0 | 25 | 619 | 87 | 20 |
| 3:67840369 | 0.87 | G/A | LOC112532082 | 0 | 1 | 25 | 617 | 72 | 10 |
| 3:67840559 | 0.86 | G/A | LOC112532082 | 0 | 1 | 25 | 626 | 80 | 11 |
| 3:67840938 | 0.82 | C/T | LOC112532082 | 0 | 1 | 24 | 602 | 108 | 15 |
| 3:67841020 | 0.56 | A/G | LOC112532082 | 0 | 0 | 25 | 408 | 217 | 95 |
| 3:67841283 | 0.72 | G/A | LOC112532082 | 0 | 0 | 26 | 518 | 154 | 40 |
| 3:67841924 | 0.88 | T/C | LOC112532082 | 0 | 1 | 25 | 643 | 71 | 11 |
| 3:67842808 | 0.86 | G/A | LOC112532082 | 0 | 0 | 26 | 625 | 81 | 17 |
| 3:67842862 | 0.68 | C/A | LOC112532082 | 0 | 0 | 26 | 496 | 153 | 64 |
| 3:67843363 | 0.53 | C/T | LOC112532082 | 0 | 0 | 26 | 389 | 162 | 129 |
| 3:67843980 | 0.47 | C/G | LOC112532082 | 0 | 0 | 26 | 355 | 215 | 153 |
| 3:67843999 | 0.49 | T/C | LOC112532082 | 0 | 0 | 26 | 356 | 231 | 136 |
| 3:67844381 | 0.61 | T/C | LOC112532082 | 0 | 0 | 26 | 455 | 187 | 87 |
| 3:67844477 | 0.44 | G/A | LOC112532082 | 0 | 1 | 25 | 327 | 247 | 148 |
| 3:67845864 | 0.68 | T/C | PDSS2 | 0 | 0 | 26 | 504 | 137 | 67 |
| 3:67845892 | 0.42 | G/A | PDSS2 | 0 | 0 | 26 | 285 | 248 | 165 |
| 3:67846516 | 0.69 | G/A | PDSS2 | 0 | 0 | 25 | 505 | 163 | 55 |
| 3:67846726 | 0.71 | G/A | PDSS2 | 0 | 0 | 26 | 526 | 150 | 50 |
| 3:67848755 | 0.60 | A/G | PDSS2 | 0 | 0 | 25 | 448 | 176 | 94 |
| 3:67848958 | 0.49 | C/T | PDSS2 | 0 | 0 | 26 | 365 | 187 | 148 |
| **3:67850419** | **0.99** | **C/G** | **PDSS2** | **0** | **0** | **22** | **537** | **7** | **0** |
